# Paternity of Subordinates Raises Cooperative Effort in Cichlids

**DOI:** 10.1371/journal.pone.0025673

**Published:** 2011-10-12

**Authors:** Rick Bruintjes, Danielle Bonfils, Dik Heg, Michael Taborsky

**Affiliations:** 1 Department of Behavioural Ecology, Institute of Ecology and Evolution, University of Bern, Hinterkappelen, Switzerland; 2 School of Biological Sciences, University of Bristol, Bristol, United Kingdom; 3 Institute of Social and Preventive Medicine (ISPM), University of Bern, Bern, Switzerland; Université Paris 13, France

## Abstract

**Background:**

In cooperative breeders, subordinates generally help a dominant breeding pair to raise offspring. Parentage studies have shown that in several species subordinates can participate in reproduction. This suggests an important role of direct fitness benefits for cooperation, particularly where groups contain unrelated subordinates. In this situation parentage should influence levels of cooperation. Here we combine parentage analyses and detailed behavioural observations in the field to study whether in the highly social cichlid *Neolamprologus pulcher* subordinates participate in reproduction and if so, whether and how this affects their cooperative care, controlling for the effect of kinship.

**Methodology/Principal Findings:**

We show that: (i) male subordinates gained paternity in 27.8% of all clutches and (ii) if they participated in reproduction, they sired on average 11.8% of young. Subordinate males sharing in reproduction showed more defence against experimentally presented egg predators compared to subordinates not participating in reproduction, and they tended to stay closer to the breeding shelter. No effects of relatedness between subordinates and dominants (to mid-parent, dominant female or dominant male) were detected on parentage and on helping behaviour.

**Conclusions/Significance:**

This is the first evidence in a cooperatively breeding fish species that the helping effort of male subordinates may depend on obtained paternity, which stresses the need to consider direct fitness benefits in evolutionary studies of helping behaviour.

## Introduction

Cooperative breeding, where subordinates help dominants to raise offspring, is rather widespread in vertebrates [1]–[5]. This helping behaviour has puzzled evolutionary biologists for a long time, as it is costly and often does not generate obvious fitness benefits to subordinates [6]–[8]. Several hypotheses have been proposed to explain the regulation of helping behaviour in cooperative breeders. First, the kin selection hypothesis predicts that subordinates should raise their level of help with increasing relatedness to recipients to acquire indirect fitness benefits, contingent on the relationship between benefits to recipients, costs to subordinates, and the relatedness between them [7]. If subordinates and dominants are only distantly related or unrelated, several mutually non-exclusive alternative hypotheses attempt to explain helping behaviour. (i) The prestige hypothesis proposes that subordinates may help dominants to signal their genetic quality to potential future partners [9]. (ii) The group augmentation hypothesis proposes that cooperative care is selected by beneficial effects of group size [10]. (iii) The pay-to-stay hypothesis proposes that subordinate helping serves as payment for being allowed to stay in the group [11], [12]. Finally (iv), helping subordinates may accrue current direct fitness benefits by participating in reproduction [13]–[15] (see [16] for a review of hypotheses).

The importance of current direct fitness benefits to subordinates obtained through parentage acquisition for the decision to help has been questioned, partly due to assumed monopolization of reproduction by dominants [3], [17]. However, studies of several cooperatively breeding vertebrates have found intra-group reproductive participation of subordinates (e.g. in fish: [18]; birds: [19] and mammals: [20]), which suggests a potential for direct fitness benefits of subordinates due to care of own offspring. In polyandrous birds with cooperative care, for example, a positive association has been found between receiving a share in mating - but not necessarily in parentage - and subordinate investment [21], [22], which highlights the potential importance of direct fitness benefits for the brood care effort of subordinate males. In cooperative breeders, a positive association between subordinate relatedness and helping effort has been observed in several cases (e.g. [13], [23], [24]), whereas in others this did not hold [25], [26] or the results were mixed [14], [27], [28]. As yet, a positive relationship between subordinates' *parentage* and their *helping effort* has been rarely found in cooperative breeders in the wild (but see [22], [29] for support in birds and mammals). Nevertheless, in cooperatively breeding fish, one experimental laboratory study showed that female subordinates unrelated to the dominants performed more alloparental brood care when they acquired a share in reproduction (i.e., when they were allowed to produce own clutches [30]).

In the cooperatively breeding cichlid fish *Neolamprologus pulcher*, subordinates are often distantly related or unrelated to the dominants, due to the high philopatry of subordinates and high turn-over rates of dominants, which is most pronounced in males [31], [32]. The main benefit of subordinates to stay in a territory of dominant breeders is the protection gained against predators, which is provided by the large group members [33], [34]. By participating in reproduction, subordinates face a threat of eviction [35], which may be detrimental due to the high mortality risk outside of territories [36]. In the laboratory, male subordinates unrelated to the breeding pair were found to participate in reproduction [31], [35], [37], [38]. However, their reproductive role in nature was questioned because subordinates have smaller gonads than breeders [17]. At present, data on subordinate parentage under natural conditions are lacking [39], and potential effects on subordinate helping effort are unknown.

In this study we combine parentage analysis and detailed behavioural observations to investigate if subordinates participate in reproduction in the field and if so, whether and how this affects subordinate helping behaviour. Due to low relatedness between dominants and subordinates, mature male subordinates can accrue only minor indirect fitness benefits by helping, which might provide incentives to acquire current direct fitness benefits through parentage acquisition. Therefore, we predicted that male subordinates should participate in reproduction in the field and that their helping effort should be contingent on paternity acquisition. Finally, we assessed whether relatedness between subordinates and dominants might affect parentage acquisition and helping behaviour of subordinates.

## Materials and Methods

### Ethics statement

This study made use of large cages (see below) that were accepted by all *N. pulcher* enclosed. The fish showed no signs of stress, and food intake rates were similar to *N. pulcher* outside the cages (200–400 plankton bites per 15 min) and to previously reported data [40]–[42]. At least every four days, all fish in the cages were monitored for signs of stress. This experiment was approved by the Zambian Ministry of Agriculture, Food and Fisheries and it complies with present laws of Zambia, the country where the study was performed. The study was approved by the Swiss Federal Veterinary Office Bern (licence no. 40/05).

### Study species

*Neolamprologus pulcher* is a monomorphic Lake Tanganyika cichlid occurring all around the sublittoral zone of the shores of Lake Tanganyika [43]. The fish were studied by SCUBA diving between 8–11 m depth at Kasakalawe point, Zambia (8°46.849′S, 31°04.882′E) from September to November 2005 and 2006. Individuals live in social groups consisting of a dominant breeding pair and usually 1–15 subordinates of both sexes that perform brood care, territory defence and maintenance [32], [33]. Detailed descriptions of the behaviour have been provided elsewhere [33], [44]. Groups contain on average 5 subordinate individuals >20 mm standard length (SL) [45] and the fish reach maturity at about 30–35 mm SL [46]. Dominance among group members is determined by size differences, even if small [34]. Large group members feed predominantly on zooplankton in the water column [32], [47], whereas small immature individuals also feed on benthic invertebrates within their territory [41]. In our study population, the fish use distinct stone patches for shelter and breeding, created by digging away sand [45], [48]. Predation risk is of key importance for group living in *N. pulcher*, since subordinates are protected by larger group members [34]. Subordinate relatedness towards newborn fry (i.e., beneficiaries) diminishes with age, due to high turn-over rates of dominants and high philopatry of subordinates [31], [32]. As a consequence, subordinates often help to raise non-kin broods [33], [49], which increases the productivity of dominants [33], [50] and lowers their work load [45], [51]. This service of subordinates is provided as payment or ‘rent’ for being tolerated and protected in the dominants' territory [36], [40], [42], [51]–[53]. Furthermore, group stability was shown to increase with group size [44]. Recently it has been shown that *N. pulcher* is able to recognize relatives [54]. The effect of relatedness between subordinates and dominants on helping effort has revealed mixed results in the field and in the laboratory, where relatedness between dominants and subordinates was negatively associated with helping effort in one study [28], and not associated with helping levels in another [26].

### Set-up and sampling

Group territories in our study population were mapped and marked with numbered stones. Experimental units were created by haphazardly selecting two adjacent *N. pulcher* group territories with groups composed of at least one breeding pair, one large (>37.5 mm SL) and one small subordinate (25–37.5 mm SL). We used groups with differently sized subordinates, because of demonstrated size-dependent responses to demand, and size-dependent task specialization [42], [52]. Small subordinates defend more against egg predators coming close to the breeding shelter than large subordinates, whereas the latter were shown to spend more effort with removing experimentally added sand from the breeding shelter [42]. Experimental groups comprised of 4.33±2.19 subordinates >15 mm SL (mean ± SD; range 2–8 subordinates/group, 16.0–48.5 mm SL). On average, 1.73±1.58 large subordinates (>37.5 mm SL), 1.73±1.16 small subordinates (25–37.5 mm SL) and 0.87±1.41 juveniles (15–24.5 mm SL) were present per group. A cage (2×2×2 m; aluminium frame covered with sturdy plastic net, mesh size 2.5×2.5 mm to allow free plankton flow) was placed over the selected units and all piscivores were removed [41]. Cages were used to allow allocation of parentage of all potential candidates, which proved to be difficult otherwise [55], [56]. In total 39 such units were created and 78 groups were enclosed for periods between 14 and 20 days. Before the quantitative recordings started, one to four subordinates per group were caught, sexed, measured (SL in mm, accuracy 0.5 mm) and marked by carefully excising half of a single fin ray of the dorsal fin to facilitate identification [42].

When there were free swimming fry at the end of the two week's observation periods, all fish larger than 15 mm SL present in the cage were caught with hand nets and transparent Plexiglas tubes to be sexed, measured and fin-clipped [41]. All fish larger than 30 mm (SL) were sexed by close inspection of the genital papilla. After removing the stone covering the breeding shelter, fry were caught with help of the anaesthetic eugenol (1 part eugenol dissolved in 4 parts 70% ethanol; [57]) and sampled wholly in Eppendorf vials together with all fish ≤15 mm SL. In one case during fry sampling, eggs were found and collected as well using tweezers. Above water, eggs, fry, fish ≤15 mm SL and the fin clips of larger fish were stored in 95% ethanol for future DNA analyses.

### Genotyping

Ten polymorphic microsatellite loci were used to determine parentage of all broods (see Text S1 for details on loci and microsatellite DNA markers). The software CERVUS3.0 [58] was used to assign offspring based on exclusion. When offspring could not be assigned to a known male, the minimum number of sires was estimated using the program GERUD2.0 [59]; see Text S1 for details). Pairwise relatedness (*r*) estimations between mature subordinates (>30 mm SL) and dominants were calculated with the program KINGROUP v2_090501 [60]. We calculated relatedness between: mature subordinates and dominant males, mature subordinates and dominant females, and mature subordinates and the midpoint of the dominant pair (average *r* to the dominant pair) using the KINSHIP estimator [61] and background allele frequencies calculated according to Konovalov & Heg (2008) [62].

### Behavioural observations

One large and one small subordinate were observed per group in random order three times for 10 min each, using a PVC-plate, soft pencil and a waterproof stopwatch. Observations were performed between 08:30 and 16:45 h and all behaviours were recorded in frequencies of occurrence, except for the time spent inside the breeding shelter. Once every minute the focal subordinates' height in the water column and its distance from the breeding shelter were estimated. Recorded behaviours included overt attacks, restrained aggressive displays, submissive behaviour and territory maintenance [33].

### Experimental sand addition and egg predator exposure

Every group was exposed twice to two experimental manipulations to create standardised estimates of helping propensity. In the sand addition trials, the breeding shelter was carefully half–covered with sand to induce digging behaviour, and digging frequencies of all group members were recorded for 10 min [41], [42], [53]. The 10 min recording of digging behaviour started after the first individual of the group began to dig, or after 5 min when no digging was shown until then. In the egg predator exposure trials one or four *Telmatochromis vittatus* were presented for 10 min in a clear Plexiglas presentation tube (length 15 cm, diameter 8.2 cm) at 5 cm distance from the breeding shelter entrance [41], [42]. The number of presented egg predators was increased from one in 2005 to four in 2006 to ascertain egg predator movement during presentations. We recorded all aggression against the presented *T. vittatus* from all group members, and the activity of the presented fish. For details about egg predator sizes and their activity levels see Text S1.

### Statistical analyses

Normality of distributions was analysed with the one-sample Kolmogorov-Smirnov test and all data were tested for homogeneity of variance. Means of the three observations per focal subordinate were calculated and if necessary, data were transformed using square root transformations. Normally distributed data were analysed with independent samples *t*-tests, whereas non-normally distributed data were analysed with Mann-Whitney *U*-tests.

The sand addition and egg predator exposure trials were analysed using Generalized Linear Mixed Models (GLMM), with the occurrence of participation in reproduction as a fixed effect and year as a random effect. All GLMMs allowed for unequal variances by adjusting the scaling parameter (deviance method [63]). Due to the small number of subordinates siring offspring, additional potential random effects like cage identity and date were not taken into account to avoid loss of predictive power. To assess whether relatedness might have affected these results, we analysed (1) whether relatedness (continuous factor: subordinate to dominant female, male or midpoint pair) predicted subordinate reproductive participation (Logistic Regression) and (2) whether relatedness (covariate) caused any effects of subordinate reproductive participation on helping behaviour in the above GLMMs.

Alpha was set to 0.05 throughout and all data were tested two-tailed. All statistical analyses were performed with SPSS software (version 17.0, SPSS Inc., Chicago, IL, USA).

## Results

In total 27 out of 78 groups (34.6%) produced fry during the experimental period. However, due to practical reasons broods of only 15 groups could be collected. From these 15 groups we collected 18 broods, rendering on average 16.4±9.1 fry and eggs per brood (range 4–33). From the 295 offspring analysed (258 fry and 37 eggs) we determined both parents of 276 offspring, one parent of 17 offspring, and no parent of one fry and one egg each (Table 1; Table S1). The majority of offspring (88.8%) were assigned to the dominant breeding pair; dominant females sired 99.7% of all offspring and dominant males sired 88.8%. In six out of 18 broods (33.3%) the dominant male shared paternity with other males and in five out of these six cases the extra-pair sires were assigned to subordinates of the same group (27.8% of all clutches; size range of subordinate males siring offspring: 31–41 mm SL). If male subordinates participated in reproduction, they gained on average 11.8% paternity. One fry was assigned to a dominant male from a neighbouring group, accounting for 7.7% extra-pair offspring in this brood. Taken together, in the six clutches with extra-pair paternity, on average 11.1% of the young in the brood were not sired by the dominant male (range: 6.3–22.7%). Subordinate females had never participated in reproduction. Seventeen young collected in one territory belonged to two different size classes indicating two separate broods; all four larger fry had been produced by the dominant female of a neighbouring territory and three out of these four young were fathered by a male not included in the cage population, suggesting a recent territory take-over by the current dominant pair preceding the experimental period.

**Table 1:** Offspring sired by different males.

Assigned fathers	Offspring number
**Father**	
Broods without extra-pair offspring	(*n* = 12 broods)
*Dominant male*	161
Broods with extra-pair paternity	(*n* = 6 broods)
Male group members	(*n* = 5 broods)
*Dominant male*	100
*Large subordinate*	14^a^
*Small subordinate*	1
Other male	(*n* = 4 broods)
*Dominant non-group male*	1
*Unknown male*	18^b^

Note that one egg did not amplify, thus no parentage could be assigned.

a Large male subordinates sired offspring in four broods.

b Including 14 eggs of two broods collected at a breeding shelter with two fry cohorts produced by two different females.

Relatedness between mature subordinates and dominants was low (Table 2) and comparable to previously reported data from this study population [28], [31]. No difference in relatedness (*r*) was detected between the dominant pair (midpoint *r*), the dominant male and the dominant female relatedness with subordinates siring part of the offspring versus subordinates that did not participate in reproduction (logistic regression, *n* = 13: midpoint *r* vs. siring effect: Wald χ^2^ = 0.699, *p* = 0.403; dominant male *r* vs. siring effect: Wald χ^2^ = 0.729, *p* = 0.393; dominant female *r* vs. siring effect: Wald χ^2^ = 0.514, *p* = 0.473).

**Table 2:** Pairwise relatedness (mean *r* ± SE) of mature subordinate males, subordinates females and all subordinates combined with dominant females, dominant males and midpoint dominant pair (average *r* between the dominant pair).

	*Midpoint r*	*Dominant males*	*Dominant females*
Male subordinates (*n* = 13)	0.172±0.033	0.096±0.084	0.199±0.055
Female subordinates (*n* = 16)	0.070±0.067	0.018±0.084	0.122±0.081
Subordinates combined (*n* = 39)*	0.096±0.242	0.058±0.311	0.116±0.296

*Includes *n* = 10 subordinates with unclear sex.

All subordinates used to calculate relatedness were larger than 30 mm SL (mean SL: 38.7±5.4 mm; range: 30.0–48.5 mm SL).

**Figure 1:**
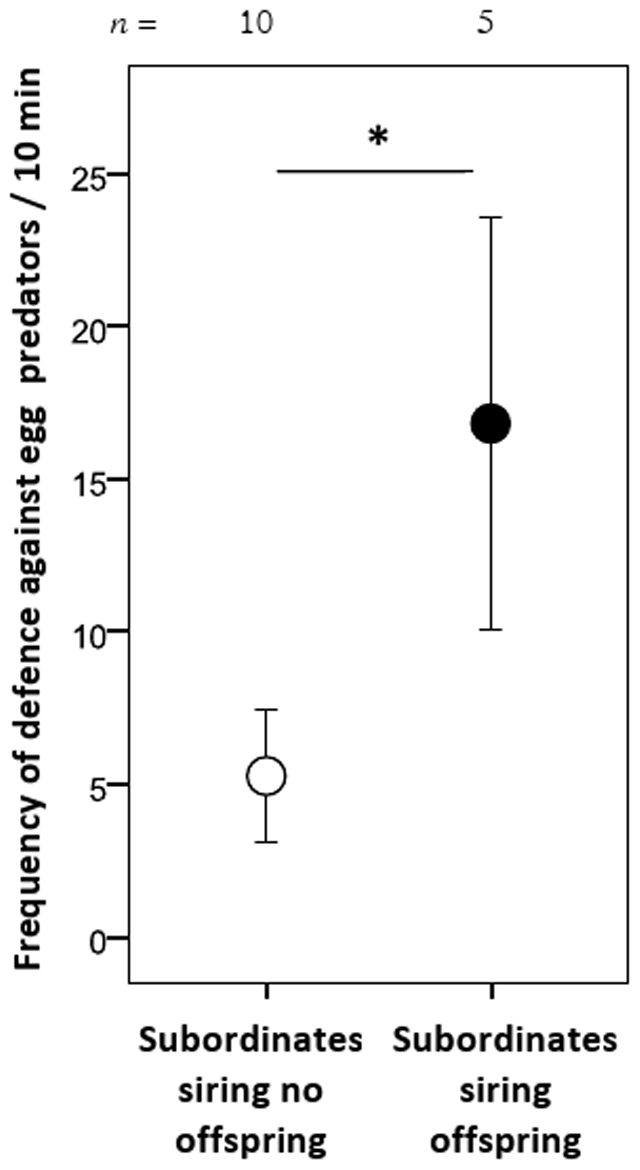
Per capita frequency of defence per 10 min for subordinates who participated in reproduction (black circle) and non-participating subordinates (open circle) against presented egg predators. Means ± SE are shown; * *p*<0.05.

In the egg predator exposure trials, subordinates who sired part of the offspring showed more defence effort against experimentally presented egg predators than same-sized subordinates that had not participated in reproduction (GLMM, *n* = 15: siring effect: Wald χ^2^ = 6.181, degrees of freedom [df] = 1, *p* = 0.013; Fig. 1); and the random effect of year was corrected for, but it was not significant (Wald χ^2^ = 2.691, df = 1, *p* = 0.101). In contrast, in the sand exposure trials no difference was found between the frequency of digging between subordinates siring offspring and those that did not (GLMM: siring effect: Wald χ^2^ = 0.110, df = 1, *p* = 0.740); and again the random effect of year was corrected for, which this time was significant, as subordinates were digging more in 2006 than in 2005 (Wald χ^2^ = 17.407, df = 1, *p*<0.001). Furthermore, these results did not change when relatedness was added as a covariate to the two GLMMs above. No effects of subordinate relatedness were detected for midpoint *r*, dominant male *r* and dominant female *r*, respectively, on defence effort against experimentally presented egg predators (0.394≤*p*≤0.922) and on digging effort in the sand exposure trials (0.233≤*p*≤0.874).

Finally, male subordinates that participated in reproduction tended to stay closer to the breeding shelter than non-participating subordinates (t-test: *t*_13_ = 1.857, *p* = 0.086), whereas no differences were found in the other behaviours tested (Table S2).

## Discussion

Our data suggest that male subordinates of *N. pulcher* participate in reproduction in the field and, if successful, they apparently raise their brood care effort accordingly. Furthermore, relatedness between subordinates and dominants did not affect the likelihood of subordinates' siring offspring. In addition, helping behaviour did not depend on relatedness between subordinates and dominants. Taken together, we can exclude kin selection as a factor explaining both subordinate parentage and helping behaviour, and the effect of subordinate parentage on their brood care effort remained significant after correcting for the (non-significant) effect of kinship. This indicates that current direct fitness benefits, such as the production of own offspring, are important for the performance and intensity of specific cooperative behaviours in subordinates of cooperatively breeding fish. In species with low relatedness between dominants and subordinates, receiving indirect fitness benefits through helping to raise offspring of relatives is improbable. This might make attempts to obtain direct fitness benefits via parentage more rewarding. In a few cooperatively breeding birds and mammals, subordinates participating in reproduction also increased their helping effort in the field [22], [29], [64], [65] and a laboratory study of *N. pulcher* revealed that female subordinates may increase alloparental care in response to their participation in reproduction [30], [38]. These data suggest that more generally, directs fitness benefits might be an important modifier of subordinate helping intensities in cooperative breeders.

Theoretical arguments suggest that in groups with multiple males, the fitness of dominant males may increase when subordinates sire only small parts of the offspring because of conflict reduction [66]. This and the increased levels of subordinate helping could select for reproductive concessions provided by dominants [67]. Previous results suggest that participation of reproduction of male [37], [38] and female subordinates in *N. pulcher* [68] is compatible with tug-of-war models of reproductive skew. Heuristic skew models, however, provide little predictive power to explain reproductive skew in cooperatively breeding fish groups due to the complexity of the mechanisms involved [69]. In *N. pulcher* it is unlikely that dominant males are in full control of reproduction, because they frantically attempt to prevent subordinates participating in spawning [34], and more dominant reproduction is lost to subordinates if multiple subordinate males are present in the group [38], [70]. Furthermore, dominant males show more aggression towards male than female subordinates [71], especially during reproductive periods [70]. This implies that male subordinates entail costs to dominant males mainly by parasitizing reproduction (cf. [35]). In compensation for these fitness costs, dominant males might benefit from increased brood care levels provided by male subordinates that have shared in reproduction. As dominant males provide virtually no help in brood care, apart from deterring large piscivores [33], [41], [42], [53], they may benefit more via increased male subordinate aid than what they lose by sharing part of reproduction. In general, the costs caused by subordinate group members have been predicted to be partly or fully compensated for by their cooperative effort if helpers pay to stay, but helping should not provide net benefits to dominants [72]. In other words, the rent helpers pay to be allowed to stay in the territory (cf. [36], [40], [42], [51]–[53], [73]) merely serves as cost compensation. This predicts that the higher the costs caused by subordinates, the more they should help, which has been supported by our data. Another incentive to increase brood care levels when successfully sharing in reproduction is the fact that some of the young benefitting from care will be own offspring. Currently, we cannot differentiate between these two potential functional causes of the positive correlation between the reproductive participation of helpers and their brood care effort.

In previous studies, extra-group paternity [55], [56] and subordinate maternity [56] had been observed under natural conditions in *N. pulcher*, however reproductive participation of male subordinates was not detected. Our results confirm the levels of male subordinate reproductive participation found in laboratory experiments [35], [37], [38]. This rather moderate reproductive participation of subordinates is difficult to detect when sample sizes per brood are small (in the previously published studies [55], [56], the mean number of sampled offspring per brood had been 3.6 and 3.9, respectively). Additionally, there is evidence that in previous studies the paternity of male subordinates may have remained undetected because of their eviction or dispersal before parentage could be determined [55]. By using large underwater cages we prevented this occurring in our study and therefore obtained genetic samples of almost all potential reproductive individuals present during offspring production. Nevertheless, one egg and one fry (0.7% of all offspring) could not be assigned to any parent and in 17 out of 295 offspring (5.8% of all offspring); one parent could not be assigned. These latter cases might have resulted at least partly from offspring production shortly before the experiment started (see Results section). Furthermore, it should be considered that we incorporated only groups with rather low numbers of subordinates in this study, which might result in an underestimation of male subordinate parentage due to a potential exponential increase of subordinate paternity with increasing numbers of male subordinates in the group [36], [62].

Reproductive participation of male subordinates in *N. pulcher* had been assumed to be ‘unlikely’ due to their relatively low investment in testis and sperm quality [17]. It remains to be tested if subordinates participating in reproduction show a higher investment in testis and sperm quality compared to the ones that did not. For instance, in the cichlid *Julidochromis ornatus* all subordinate males are likely to participate in reproduction [17], and testis mass correlates positively between dominant male breeders and their male subordinates, which suggests adjustments to the level of intragroup sperm competition [74]. Our results show that in *N. pulcher*, relatedness did not differ between the dominant pair and subordinates that sired part of the offspring and those that did not. Furthermore, when testing for effects of subordinate parentage and relatedness to dominants on subordinate defence against egg predators, only subordinate parentage showed significant effects, but not relatedness levels between subordinates and dominants. This indicates that relatedness does not strongly affect subordinate helping effort in *N. pulcher*, or at least not as strongly as subordinate parentage does.

Our results show that male subordinates sharing in reproduction tended to stay closer to the breeding shelter, which might serve as a guarding function. Previously we have reported size-dependent sharing of tasks among subordinates of this species, with small subordinates specialising in defence against egg predators [42]. Our new findings suggest that in addition to size dependence, the effort of male subordinates in brood care and protection may also depend on their participation in reproduction. In banded mongooses, especially male subordinates contribute more to guarding during times of high energy expenditure and the survival rates of young increases with the number of guards [75]. However, in contrast to *N. pulcher*, banded mongoose yearling non-breeding males tended to make higher individual contributions to the care of pups than yearling breeding males that may have successfully participated in offspring production [65].

Digging behaviour seems to be less flexible than defence in *N. pulcher*, as the removal of experimentally added sand from the breeding shelter was not related to the subordinates' share in reproduction. This confirms previous results of similar sand addition trials revealing little plasticity of large subordinates in response to varying digging demands. For instance, no difference in digging intensity was found *among* large subordinates between: (i) low and high neighbour densities simulating variation in space competition [52]; (ii) natural and reduced food conditions [41]; (iii) isolated and group living individuals [76]; (iv) low and high densities of egg predators [42]; and (v) differences in reproductive status of groups, i.e. with or without free-swimming fry (Bruintjes R, Louter M & Taborsky M, unpubl. data).

In conclusion, our data show that in cooperatively breeding cichlids male subordinates can gain parentage in the field, and that this might affect their effort spent on specific helping behaviours. Our results stress that current direct fitness benefits (participation in reproduction) might be of importance in modifying subordinate helping effort in cooperative breeders.

## Supporting Information

Table S1 **Parentage of 18 broods collected from 15 groups in the field.**
(DOCX)

Table S2 **Behavioural comparisons of subordinates with and without parentage.** The table shows all focal behaviours which were tested with independent sample *t*-tests, except for submissiveness, which was tested with a Mann-Whitney U-test. 0.05<*p*-values<0.10 are underlined.
(DOCX)

Text S1 **This supplementary text contains additional details on genotyping, parentage assignment and egg predator presentation trials.**
(DOCX)

## References

[1] Stacey PB, Koenig WD (1990). Cooperative breeding in birds: Long-term studies of ecology and behavior.

[2] Taborsky M (1994). Sneakers, satellites and helpers: parasitic and cooperative behavior in fish reproduction.. Advances in the Study of Behavior.

[3] Cockburn A (1998). Evolution of helping behavior in cooperatively breeding birds.. Annual Review of Ecology and Systematics.

[4] Cochran GR, Solomon NG (2000). Effects of food supplementation on the social organization of prairie voles (*Microtus ochrogaster*).. Journal of Mammalogy.

[5] Heg D, Bachar Z (2006). Cooperative breeding in the lake tanganyika cichlid *Julidochromis ornatus*.. Environmental Biology of Fishes.

[6] Hamilton WD (1963). The evolution of altruistic behavior.. American Naturalist.

[7] Hamilton WD (1964). The genetical evolution of social behaviour I & II.. Journal of Theoretical Biology.

[8] Lehmann L, Keller L (2006). The evolution of cooperation and altruism - a general framework and a classification of models.. Journal of Evolutionary Biology.

[9] Zahavi A (1995). Altruism as a handicap - the limitations of kin selection and reciprocity.. Journal of Avian Biology.

[10] Kokko H, Johnstone RA, Clutton-Brock TH (2001). The evolution of cooperative breeding through group augmentation.. Proceedings of the Royal Society of London Series B-Biological Sciences.

[11] Gaston AJ (1978). Evolution of group territorial behavior and cooperative breeding.. American Naturalist.

[12] Kokko H, Johnstone RA, Wright J (2002). The evolution of parental and alloparental effort in cooperatively breeding groups: when should helpers pay to stay?. Behavioral Ecology.

[13] Magrath RD, Whittingham LA (1997). Subordinate males are more likely to help if unrelated to the breeding female in cooperatively breeding white-browed scrubwrens.. Behavioral Ecology and Sociobiology.

[14] Dickinson JL (2004). A test of the importance of direct and indirect fitness benefits for helping decisions in western bluebirds.. Behavioral Ecology.

[15] Skubic E, Taborsky M, McNamara JM, Houston AI (2004). When to parasitize? A dynamic optimization model of reproductive strategies in a cooperative breeder.. Journal of Theoretical Biology.

[16] Bergmuller R, Johnstone RA, Russell AF, Bshary R (2007). Integrating cooperative breeding into theoretical concepts of cooperation.. Behavioural Processes.

[17] Fitzpatrick JL, Desjardins JK, Stiver KA, Montgomerie R, Balshine S (2006). Male reproductive suppression in the cooperatively breeding fish *Neolamprologus pulcher*.. Behavioral Ecology.

[18] Awata S, Munehara H, Kohda M (2005). Social system and reproduction of helpers in a cooperatively breeding cichlid fish (*Julidochromis ornatus*) in Lake Tanganyika: field observations and parentage analyses.. Behavioral Ecology and Sociobiology.

[19] Whittingham LA, Dunn PO, Magrath RD (1997). Relatedness, polyandry and extra-group paternity in the cooperatively-breeding white-browed scrubwren (*Sericornis frontalis*).. Behavioral Ecology and Sociobiology.

[20] Goossens B, Graziani L, Waits LP, Farand E, Magnolon S (1998). Extra-pair paternity in the monogamous Alpine marmot revealed by nuclear DNA microsatellite analysis.. Behavioral Ecology and Sociobiology.

[21] Davies NB (1992). Dunnock Behaviour and Social Evolution.

[22] Hartley IR, Davies NB, Hatchwell BJ, Desrochers A, Nebel D (1995). The Polygynandrous Mating System of the Alpine Accentor, *Prunella-Collaris*. II. Multiple Paternity and Parental Effort.. Animal Behaviour.

[23] Nam KB, Simeoni M, Sharp SP, Hatchwell BJ (2010). Kinship affects investment by helpers in a cooperatively breeding bird.. Proceedings of the Royal Society B-Biological Sciences.

[24] Kingma SA, Hall ML, Peters A (2011). Multiple Benefits Drive Helping Behavior in a Cooperatively Breeding Bird: An Integrated Analysis.. American Naturalist.

[25] Armitage KB, Schwartz OA (2000). Social enhancement of fitness in yellow-bellied marmots.. Proceedings of the National Academy of Sciences of the United States of America.

[26] Le Vin AL, Mable BK, Taborsky M, Heg D, Arnold KE (2011). Individual variation in helping in a cooperative breeder: relatedness versus behavioural type.. Animal Behaviour.

[27] Wright J, McDonald PG, te Marvelde L, Kazem AJN, Bishop CM (2010). Helping effort increases with relatedness in bell miners, but ‘unrelated’ helpers of both sexes still provide substantial care.. Proceedings of the Royal Society B-Biological Sciences.

[28] Stiver KA, Dierkes P, Taborsky M, Gibbs HL, Balshine S (2005). Relatedness and helping in fish: examining the theoretical predictions.. Proceedings of the Royal Society B-Biological Sciences.

[29] Canestrari D, Marcos JM, Baglione V (2005). Effect of parentage and relatedness on the individual contribution to cooperative chick care in carrion crows *Corvus corone corone*.. Behavioral Ecology and Sociobiology.

[30] Heg D, Jutzeler E, Mitchell JS, Hamilton IM (2009). Helpful Female Subordinate Cichlids Are More Likely to Reproduce.. Plos One.

[31] Dierkes P, Heg D, Skubic E, Taborsky M, Achmann R (2005). Genetic relatedness in groups is sex-specific and declines with age of helpers in a cooperatively breeding cichlid.. Ecology Letters.

[32] Taborsky M, Limberger D (1981). Helpers in fish.. Behavioral Ecology and Sociobiology.

[33] Taborsky M (1984). Broodcare helpers in the cichlid fish *Lamprologus brichardi*: their costs and benefits.. Animal Behaviour.

[34] Heg D, Bachar Z, Brouwer L, Taborsky M (2004). Predation risk is an ecological constraint for helper dispersal in a cooperatively breeding cichlid.. Proceedings of the Royal Society of London Series B-Biological Sciences.

[35] Dierkes P, Taborsky M, Kohler U (1999). Reproductive parasitism of broodcare helpers in a cooperatively breeding fish.. Behavioral Ecology.

[36] Taborsky M (1985). Breeder-helper conflict in a cichlid fish with broodcare helpers: an experimental analysis.. Behaviour.

[37] Heg D, Bergmüller R, Bonfils D, Otti O, Bachar Z (2006). Cichlids do not adjust reproductive skew to the availability of independent breeding options.. Behavioral Ecology.

[38] Heg D, Jutzeler E, Bonfils D, Mitchell JS (2008). Group composition affects male reproductive partitioning in a cooperatively breeding cichlid.. Molecular Ecology.

[39] Wong M, Balshine S (2011). The evolution of cooperative breeding in the African cichlid fish, *Neolamprologus pulcher*.. Biological Reviews.

[40] Balshine-Earn S, Neat FC, Reid H, Taborsky M (1998). Paying to stay or paying to breed? Field evidence for direct benefits of helping behavior in a cooperatively breeding fish.. Behavioral Ecology.

[41] Bruintjes R, Hekman R, Taborsky M (2010). Experimental global food reduction raises resource acquisition costs of brood care helpers and reduces their helping effort.. Functional Ecology.

[42] Bruintjes R, Taborsky M (2011). Size dependent task specialization in a cooperative cichlid in response to experimental variation of demand.. Animal Behaviour.

[43] Duftner N, Sefc KM, Koblmuller S, Salzburger W, Taborsky M (2007). Parallel evolution of facial stripe patterns in the *Neolamprologus brichardi*/*pulcher* species complex endemic to Lake Tanganyika.. Molecular Phylogenetics and Evolution.

[44] Heg D, Brouwer L, Bachar Z, Taborsky M (2005). Large group size yields group stability in the cooperatively breeding cichlid *Neolamprologus pulcher*.. Behaviour.

[45] Balshine S, Leach B, Neat F, Reid H, Taborsky M (2001). Correlates of group size in a cooperatively breeding cichlid fish (Neolamprologus pulcher).. Behavioral Ecology and Sociobiology.

[46] Heg D, Rothenberger S, Schürch R (2011). Habitat saturation, benefits of philopatry, relatedness, and the extent of co-operative breeding in a cichlid.. Behavioral Ecology.

[47] Gashagaza MM, Nagoshi M (1986). Comparative study on the food habits of six species of Lamprologus (Osteichthyes: Cichlidae).. African Study Monographs.

[48] Heg D, Heg-Bachar Z, Brouwer L, Taborsky M (2008). Experimentally induced helper dispersal in colonially breeding cooperative cichlids.. Environmental Biology of Fishes.

[49] Brouwer L, Heg D, Taborsky M (2005). Experimental evidence for helper effects in a cooperatively breeding cichlid.. Behavioral Ecology.

[50] Taborsky B, Skubic E, Bruintjes R (2007). Mothers adjust egg size to helper number in a cooperatively breeding cichlid.. Behavioral Ecology.

[51] Bergmüller R, Taborsky M (2005). Experimental manipulation of helping in a cooperative breeder: helpers ‘pay to stay’ by pre-emptive appeasement.. Animal Behaviour.

[52] Bruintjes R, Taborsky M (2008). Helpers in a cooperative breeder pay a high price to stay: effects of demand, helper size and sex.. Animal Behaviour.

[53] Heg D, Taborsky M (2010). Helper Response to Experimentally Manipulated Predation Risk in the Cooperatively Breeding Cichlid *Neolamprologus pulcher*.. Plos One.

[54] Le Vin AL, Mable BK, Arnold KE (2010). Kin recognition via phenotype matching in a cooperatively breeding cichlid, Neolamprologus pulcher.. Animal Behaviour.

[55] Dierkes P, Taborsky M, Achmann R (2008). Multiple paternity in the cooperatively breeding fish *Neolamprologus pulcher*.. Behavioral Ecology and Sociobiology.

[56] Stiver KA, Fitzpatrick JL, Desjardins JK, Balshine S (2009). Mixed parentage in *Neolamprologus pulcher* groups.. Journal of Fish Biology.

[57] Kreiberg H, Ostrander GK (2000). Stress and anesthesia.. The Laboratory Fish.

[58] Kalinowski ST, Taper ML, Marshall TC (2007). Revising how the computer program CERVUS accommodates genotyping error increases success in paternity assignment.. Molecular Ecology.

[59] Jones AG (2005). GERUD 2.0: a computer program for the reconstruction of parental genotypes from half-sib progeny arrays with known or unknown parents.. Molecular Ecology Notes.

[60] Konovalov DA, Manning C, Henshaw MT (2004). KINGROUP: a program for pedigree relationship reconstruction and kin group assignments using genetic markers.. Molecular Ecology Notes.

[61] Queller DC, Goodnight KF (1989). Estimating relatedness using genetic markers.. Evolution.

[62] Konovalov DA, Heg D (2008). A maximum-likelihood relatedness estimator allowing for negative relatedness values.. Molecular Ecology Resources.

[63] Norusis MJ (2010). PASW Statistics 18 Advanced Statistical Procedures Companion.

[64] Whittingham LA, Dunn PO (1998). Male parental effort and paternity in a variable mating system.. Animal Behaviour.

[65] Gilchrist JS, Russell AF (2007). Who cares? Individual contributions to pup care by breeders vs non-breeders in the cooperatively breeding banded mongoose (*Mungos mungo*).. Behavioral Ecology and Sociobiology.

[66] Houston AI, Gasson CE, McNamara JM (1997). Female choice of matings to maximize parental care.. Proceedings of the Royal Society of London Series B-Biological Sciences.

[67] Keller L, Reeve HK (1994). Partitioning of Reproduction in Animal Societies.. Trends in Ecology & Evolution.

[68] Heg D, Hamilton IM (2008). Tug-of-war over reproduction in a cooperatively breeding cichlid.. Behavioral Ecology and Sociobiology.

[69] Taborsky M, Hager R, Jones CB (2009). Reproductive skew in cooperative fish groups: virtue and limitations of alternative modeling approaches.. Reproductive Skew in Vertebrates: Proximate and Ulitimate Causes.

[70] Mitchell JS, Jutzeler E, Heg D, Taborsky M (2009). Gender Differences in the Costs that Subordinate Group Members Impose on Dominant Males in a Cooperative Breeder.. Ethology.

[71] Mitchell JS, Jutzeler E, Heg D, Taborsky M (2009). Dominant members of cooperatively-breeding groups adjust their behaviour in response to the sexes of their subordinates.. Behaviour.

[72] Hamilton IM, Taborsky M (2005). Unrelated helpers will not fully compensate for costs imposed on breeders when they pay to stay.. Proceedings of the Royal Society B-Biological Sciences.

[73] Bergmüller R, Heg D, Taborsky M (2005). Helpers in a cooperatively breeding cichlid stay and pay or disperse and breed, depending on ecological constraints.. Proceedings of the Royal Society B-Biological Sciences.

[74] Awata S, Heg D, Munehara H, Kohda M (2006). Testis size depends on social status and the presence of male helpers in the cooperatively breeding cichlid *Julidochromis ornatus*.. Behavioral Ecology.

[75] Cant MA (2003). Patterns of helping effort in co-operatively breeding banded mongooses (*Mungos mungo*).. Journal of Zoology.

[76] Schürch R, Heg D (2010). Life history and behavioral type in the highly social cichlid *Neolamprologus pulcher*.. Behavioral Ecology.

