# Supplementary material for: Paternity of Subordinates Raises Cooperative Effort in Cichlids
Source: PLoS One. 2011 Oct 12;6(10):e25673. doi: 10.1371/journal.pone.0025673 (PMC3192049; doi:10.1371/journal.pone.0025673)
Supplement: Table S1 — Parentage of 18 broods collected from 15 groups in the field. (DOCX) [file pone.0025673.s001.docx]

**Supplementary Table S1** *for* Paternity of subordinates raises cooperative effort in cichlids, Bruintjes *et al.* 2011

**Table S1.** Parentage of 18 broods collected from 15 groups in the field.

| **Group ID** | **Brood size** | **Fry**  **(f)or eggs (e)** | **% Young of D**♂ | **% Young of D**♀ | **Known individu-als siring offspring (not D**♂) | **# Young sired by known** ♂♂ | **% of reproduc-tive success of G SS**♂**, G LS**♂ **and dif D**♂ | **Un-known individu-als siring offspring** | **# Young sired by 1 un-known parent** | **% of repro-ductive success of U**♂ | **% Young with two un-known parents** | **Ha-rem**  **yes / no** | **Sam-pling year** | | **Mini-mum # of fathers^a^** | **#**  **Mature S**♂ **(>30 mm) per group** | **#**  **Mature S**♀ **(>30 mm) per group** |
| --- | --- | --- | --- | --- | --- | --- | --- | --- | --- | --- | --- | --- | --- | --- | --- | --- | --- |
| K31 | 25 | f | 92.0 | 100.0 | G LS♂ | 2 | 8.0 |  | 0 | 0.0 | 0.0 | no | ´05 | | 2 | 1 | 1 |
| K34 | 16 | f | 81.3 | 100.0 | G SS♂ | 1 | 6.3 | U♂ | 2 | 12.5 | 0.0 | yes | ´05 | | 3 | 2 | 0 |
| C32 | 8 | f | 100.0 | 100.0 |  | 0 | 0.0 |  | 0 | 0.0 | 0.0 | no | ´05 | | 1 | 0 | 1 |
| L108 | 9 | f | 100.0 | 100.0 |  | 0 | 0.0 |  | 0 | 0.0 | 0.0 | yes | ´05 | | 1 | 3 | 4 |
| L108A | 37 | e | 80.6 | 97.2 | G LS♂ | 4 | 11.1 | U♂ | 2 | 5.4 | 2.8 | yes | ´05 | | 3^b^ | 3 | 4 |
| C27 | 13 | f | 15.4 | 100.0 | dif D♂ | 1 | 7.7 | U♂ | 10 | 76.9 | 0.0 | yes | ´05 | | 3 | 0 | 1 |
| C27A | 4 | f | 25.0 | 100.0 |  | 0 | 0.0 | U♂ | 3 | 75.0 | 0.0 | Yes | ´05 | | 2 | 0 | 0 |
| K7 | 5 | f | 100.0 | 100.0 |  | 0 | 0.0 |  | 0 | 0.0 | 0.0 | yes | ´05 | | 1 | 1 | 0 |
| K16 | 13 | f | 100.0 | 100.0 |  | 0 | 0.0 |  | 0 | 0.0 | 0.0 | no | ´05 | | 1 | 0 | 2 |
| S87 | 25 | f | 100.0 | 100.0 |  | 0 | 0.0 |  | 0 | 0.0 | 0.0 | yes | ´06 | | 1 | 0 | 0 |
| S87A | 18 | f | 100.0 | 100.0 |  | 0 | 0.0 |  | 0 | 0.0 | 0.0 | yes | ´06 | | 1 | 0 | 0 |
| S88 | 33 | f | 100.0 | 100.0 |  | 0 | 0.0 |  | 0 | 0.0 | 0.0 | yes | ´06 | | 1 | 1 | 2 |
| S81 | 10 | f | 100.0 | 100.0 |  | 0 | 0.0 |  | 0 | 0.0 | 0.0 | no | ´06 | | 1 | 1 | 0 |
| S97 | 16 | f | 100.0 | 100.0 |  | 0 | 0.0 |  | 0 | 0.0 | 0.0 | no | ´06 | | 1 | 1 | 1 |
| S108 | 10 | f | 100.0 | 100.0 |  | 0 | 0.0 |  | 0 | 0.0 | 0.0 | no | ´06 | | 1 | 0 | 1 |
| S149 | 13 | f | 100.0 | 100.0 |  | 0 | 0.0 |  | 0 | 0.0 | 0.0 | no | ´06 | | 1 | 0 | 2 |
| S113 | 22 | f | 77.3 | 100.0 | G LS♂ | 5 | 22.7 |  | 0 | 0.0 | 0.0 | no | ´06 | | 2 | 1 | 1 |
| S121 | 18 | f | 88.9 | 100.0 | G LS♂ | 2 | 11.1 |  | 0 | 0.0 | 0.0 | no | ´06 | | 2 | 1 | 1 |
| **Total:** | 295 |  | **Mean reproductive success of non-dominant group males siring offspring :** | | | | 11.1 |  |  |  |  | |  |  |  |  |  |

D♂: Dominant male; D♀: Dominant female, G LS♂; Male large subordinate of own group; G SS♂: Male small subordinate of own group; dif D♂: Dominant male from different group; U♂: Unknown male (probably not contained in the cage); S♂: male subordinate; S♀: female subordinate.

^a^ Minimum number of fathers in broods was estimated with help of GERUD 2.0.

^b^ The minimum number of fathers was calculated for 27 eggs only, since the alleles of ten eggs did not amplify and thus could not be analysed.
