# Supplementary material for: Paternity of Subordinates Raises Cooperative Effort in Cichlids
Source: PLoS One. 2011 Oct 12;6(10):e25673. doi: 10.1371/journal.pone.0025673 (PMC3192049; doi:10.1371/journal.pone.0025673)
Supplement: Table S2 — Behavioural comparisons of subordinates with and without parentage. The table shows all focal behaviours which were tested with independent sample t-tests, except for submissiveness, which was tested with a Mann-Whitney U-test. 0.05<p-values<0.10 are underlined. (DOCX) [file pone.0025673.s002.docx]

**Supplementary Table S2** *for* Paternity of subordinates raises cooperative effort in cichlids, Bruintjes *et al.* 2011

**Table S2.** Behavioural comparisons of subordinates with and without parentage. The table shows all focal behaviours which were tested with independent sample *t*-tests, except for submissiveness, which was tested with a Mann-Whitney U-test. 0.05 < *p*-values < 0.10 are underlined.

|  | Statistic | | (*n* = 15) | Significance |
| --- | --- | --- | --- | --- |
|  | *t_13_* | |  | *p* |
| Distance to shelter | 1.857 | |  | 0.086 |
| Height in water column  Number of shelter visits | 0.989  -0.807 | |  | 0.341  0.434 |
| Total time in shelter | 0.913 |  | | 0.378 |
|  |  |  | |  |
| Submissiveness | *U*  19.0 |  | | *p*  0.403 |
|  |  |  | |  |
