# Supplementary material for: Paternity of Subordinates Raises Cooperative Effort in Cichlids
Source: PLoS One. 2011 Oct 12;6(10):e25673. doi: 10.1371/journal.pone.0025673 (PMC3192049; doi:10.1371/journal.pone.0025673)
Supplement: Text S1 — This supplementary text contains additional details on genotyping, parentage assignment and egg predator presentation trials. (DOCX) [file pone.0025673.s003.docx]

**Supplementary Text S1** *for* Paternity of subordinates raises cooperative effort in cichlids, Bruintjes *et al.* 2011

This supplementary text contains additional details on genotyping, parentage assignment and egg predator presentation trials.

## Genotyping

Ten polymorphic microsatellite loci were used to determine parentage of the 18 broods (loci NP007, NP773, UL12: [1]; Pzeb4: [2]; TmoM11, TmoM13, TmoM25, TmoM27; [3]; UME003: [4]; UNH154: [5]. All loci had 2-32 alleles, with a mean of 16.9 alleles per locus.

Genomic DNA was extracted from ethanol-preserved fin clip samples of dominants and subordinates, whole larvae or eggs using a manual 96 well format DNA extraction protocol on the basis of a magnetic separation technique. Tissue lysis was carried out in a Lysis-Buffer containing Nuclei Lysis Solution (Promega), 0.5M EDTA and Proteinase K according to the Wizard Genomic DNA Isolation Protocol (Technical Manual No. TM050, Promega). DNA was captured in solution by adding Paramagnetic Particles (MagneSil® Blue, Promega; [6] and washed 2-3 times with 80% ethanol with the aid of a magnetic separator (MagnaBot®96 Magnetic Separation Device, Promega) to eliminate residual contaminants. Finally, genomic DNA was eluted directly from the Paramagnetic Particles with 50-100µl of Nuclease Free Water and kept at -20°C until further analyses.

For polymerase chain reaction (PCR) amplification, all ten microsatellite primer pairs were multiplexed in one PCR reaction using the QIAGEN® Multiplex PCR Kit (Qiagen). PCR reactions were carried out in a 10µl volume containing 1-2ul of the genomic DNA, 1x QIAGEN® Multiplex PCR Master Mix (consisting of QIAGEN® Multiplex PCR buffer with a final concentration of 3 mM MgCl_2_, dNTP mix and HotStar *taq* DNA polymerase) and 2 µM of each locus-specific 5’ fluorescent labelled forward primer (fluorescent dyes were 6-FAM, HEX, NED and PET) and non labelled reverse primer. Eight of the ten reverse primers (NP007, NP773, ULI2, UME003, TmoM11, TmoM13, TmoM25 and TmoM27) were additionally modified by placing the nucleotide sequence GTTTCTT on the 5’ end [7]. This reverse-primer tailing results in nearly 100% adenylation of the 3’ end of the forward strands, thereby facilitating accurate genotyping as a result of consistent allele calls.

Amplification was achieved in a GeneAmp® 9700 Thermocycler (Applied Biosystems), using the following sequence of cycling parameters: 15 min at 95°C; 35 cycles at 94°C for 30 sec, 57°C for 3 min, and 72°C for 15 min, followed by a final 15 min extension at 72°C. Fluorescent PCR fragments were visualized by capillary electrophoresis on an ABI PRISM® 3100 Genetic Analyzer and analyzed by the GeneMapper® Analysis Software version 3.7 (Applied Biosystems).

## Parentage assignment

Assignment of parentage was primarily based on exclusion, with allowance for one genetic mismatch in the parentage analysis to take potential errors arising from variation in DNA sequence, DNA quality and scoring error into account [8]. Parentage was assigned using the software CERVUS 3.0 [9] and potential parents for each offspring were the dominant pair, its subordinates and all other sexually mature individuals present in the same cage. When offspring could not be assigned to a known male, the software GERUD2.0 was used to estimate the minimum number of sires [10]. If a group dominant and another individual could not be excluded as a parent, parentage was assigned to the dominant group member except when the LOD score (the natural log of the overall likelihood ratio of parentage for a candidate parent) to the other potential parent exceeded that of the dominant group member (cf. [11]). In one instance, one egg that amplified for five of the six loci was assigned to having been sired by the dominant female that sired this whole brood.

## **Egg predator presentation trials**

In 2005, the average size of the presented *T. vittatus* was 32.4 ± 3.1 mm (mean SL ± SE; *n* = 12 different individuals) and in 2006 it was 31.7 ± 2.0 mm (*n* = 20 different individuals). During the egg predator presentations we noted the activity level of the exposed intruder(s) on a scale from 0 to 5 (0 = no activity, 5 = very active). Neither *T. vittatus* size nor their recorded activity differed between years (size: *t*-test, *t*_30_ = -0.835, *P* = 0.410; activity: Mann-Whitney *U*-test, *z* = -0.830, *P* = 0.406).

# **References**

1. Schliewen U, Rassmann K, Markmann M, Markert J, Kocher T, Tautz D (2001) Genetic and ecological divergence of a monophyletic cichlid species pair under fully sympatric conditions in Lake Ejagham, Cameroon. Mol Ecol 10: 1471-1488.

2. VanOppen MJH, Rico C, Deutsch JC, Turner GF, Hewitt GM (1997) Isolation and characterization of microsatellite loci in the cichlid fish Pseudotropheus zebra. Mol Ecol 6: 387-388.

3. Zardoya R, Vollmer DM, Craddock C, Streelman JT, Karl S, Meyer A (1996) Evolutionary conservation of microsatellite flanking regions and their use in resolving the phylogeny of cichlid fishes (Pisces: Perciformes). Proc R Soc Lond B 263: 1589-1598.

4. Parker A, Kornfield I (1996) Polygynandry in Pseudotropheus zebra, a cichlid fish from Lake Malawi. Environ Biol Fish 47: 345-352.

5. Lee WJ, Kocher TD (1996) Microsatellite DNA markers for genetic mapping in Oreochromis niloticus. J Fish Biol 49: 169-171.

6. White D, Butler B, Creswell D, Smith C (1998) MagneSil^TM^ Paramagnetic Particles: Novel Magnetics for DNA Purification. Promega Notes [69]. Ref Type: Online Source.

7. Brownstein MJ, Carpten JD, Smith JR (1996) Modulation of non-templated nucleotide addition by tag DNA polymerase: Primer modifications that facilitate genotyping. Biotechniques 20: 1004-1010.

8. Pompanon F, Bonin A, Bellemain E, Taberlet P (2005) Genotyping errors: Causes, consequences and solutions. Nat Rev Gen 6: 847-859.

9. Kalinowski ST, Taper ML, Marshall TC (2007) Revising how the computer program CERVUS accommodates genotyping error increases success in paternity assignment. Mol Ecol 16: 1099-1106.

10. Jones AG (2005) GERUD 2.0: a computer program for the reconstruction of parental genotypes from half-sib progeny arrays with known or unknown parents. Mol Ecol Notes 5: 708-711.

11. Stiver KA, Fitzpatrick JL, Desjardins JK, Balshine S (2009) Mixed parentage in Neolamprologus pulcher groups. J Fish Biol 74: 1129-1135.

12. Bruintjes R, Taborsky M (2011) Size dependent task specialization in a cooperative cichlid in response to experimental variation of demand. Anim Behav 81: 387-394.
